# Implementation of lasting changes to sustainability in Interventional Radiology is a top-down governance challenge

**DOI:** 10.1186/s42155-023-00371-0

**Published:** 2023-04-01

**Authors:** Warren Clements

**Affiliations:** 1 Department of Radiology, Alfred Health, 55 Commercial Road, Melbourne, VIC 3004 Australia; 2 Department of Surgery, Central Clinical School, Monash University, Melbourne, Australia; 3 National Trauma Research Institute, Melbourne, Australia

**Keywords:** Recycling, Waste, Sustainability, Emissions, Energy

The world is still beginning to awaken to the effect of carbon emissions on climate change and its associated health and economic consequences. Hospitals have been shown to be a major contributor to climate change (Slanetz et al. 2022), estimated to contribute approximately 7000 tonnes of waste per day through a combination of energy consumption and waste generation (Shum et al. 2022). Radiology departments are a major component of this, particularly CT and MRI scanners (Martin et al. 2022; Heye et al. 2020).

Interventional Radiologists (IRs) are also finally beginning to identify and address this issue, both in quantification of waste but also steps to address change at an individual-level. Chua et al. quantified greenhouse gas emissions in their recent study of 98 Interventional Radiology procedures, estimating 23,500 kg of carbon dioxide were emitted during their study (Chua et al. 2021). The biggest contributor was energy consumption through climate-controlled air conditioning, whilst the second biggest contributor was production and transportation of disposable surgical items (Chua et al. 2021). Some waste material is deliberately single use to reduce the risk of infection. However, a recent study showed that an astonishing 54.8% of IR product packaging was waste (Clements et al. 2020). While 76% of this waste was potentially recyclable, reduction of unnecessary waste is still preferred to recycling (Clements et al. 2020).

De Reeder et al. discuss barriers and enablers of change in their recent manuscript, and many IRs in the Netherlands are motivated to change practice (Reeder et al. 2023), likely to be true for many other countries as well. The cliché of “reduce, reuse, recycle” is as important now as it has always been. We should reduce our usage of waste products by only opening items we are using and/or trying to avoid excessive use of disposable equipment (Vyval et al. 2021). We should reduce our power consumption and turn off unused electrical items (Shum et al. 2022). We should be sorting our waste and recycling non-hazardous material in their appropriate bin (Clements et al. 2020; Brassil and Torreggiani 2019). But, I know that all IRs already know this and are contributing as they best can (Flowers 2020).

What are the next steps that we can do in IR? The focus of group advocacy should start at a high-level targeted at healthcare departments in government, and major influential health organisations. Advocacy for sustainability in IR is best achieved in numbers through major societies such as the Cardiovascular and Interventional Society of Europe (CIRSE) and the Society of Interventional Radiology (SIR). It is interesting to note that at the time of writing, neither CIRSE nor SIR have a published position statement on sustainability in IR. But, asking CIRSE or SIR to “fix it” on their own also is not sufficient. Sustainability requires volunteers to give their time for the cause—both to major societies but also to their local network. Publication of supportive IR sustainability guidelines and statements will allow local IRs to use the broader work of our societies to campaign for changes at their own institution. We should also partner with existing established climate change organisations and lobbyists as has been done in other industries.

Ultimately, change in healthcare comes from a well-trodden process such as the Kotter model, and changes must be based on healthcare science and underpinned by data (Steele et al. 2012). While IRs are now beginning the process of generating some data on the topic of waste and sustainability (Chua et al. 2021; Clements et al. 2020; Reeder et al. 2023; Vyval et al. 2021), it is still dwarfed by existing and ongoing research on clinical practice, and there simply aren’t enough clinicians focussing their research attention on this important topic.

Change also requires individuals to articulate plans through leadership and not just managing the status quo. I would encourage all IRs to identify “climate leaders” in their team and give them the bandwidth to prosper in both a research and governance manner. These leaders may be anyone on their journey to IR, and not just those in senior management positions who often simply perpetuate existing organisational goals (Clements 2023).

However, individual changes and to some degree even changes from a small stakeholder group are not going to be enough on their own. For healthcare systems to make meaningful and lasting change, there needs to be a top-down approach. This must include hospital executive management engaging in sustainability or “green” teams through focus on making change to existing practices, and dictate that sustainability is built into frameworks for all change of infrastructure and practice moving forward. This will help embed it within organisational culture and make it virtually impossible for a hospital and its employees to avoid sustainable practice. In assessing the effects of these changes, key performance metrics need to be developed and made publicly available, for example volume of waste and carbon dioxide emissions. This will hold hospitals and health networks accountable and allow comparison between sites to encourage competition, and even potentially shame underperforming networks. If governments so desire, hospital remuneration could even be tied in to meeting sustainability targets.

We are already beyond the ideal time to start this process. I would suggest that IRs start by considering and reading the studies in this issue, and in the attached references. IRs should focus attention on sustainability-related research and drive data on sustainability through minimisation of waste and energy consumption, but also on systems that lead to positive changes. Once there is data, we can advocate for top-down change through partnering with CIRSE and other established stakeholders and lobbyists. I look forward to reading about data-driven sustainability improvements through the pages of CVIR Endovascular in the coming years.

## Data Availability

N/A.
